# Application of Xanthan Gum and Hyaluronic Acid as Dermal Foam Stabilizers

**DOI:** 10.3390/gels8070413

**Published:** 2022-06-30

**Authors:** Fanni Falusi, Szilvia Berkó, Anita Kovács, Mária Budai-Szűcs

**Affiliations:** Institute of Pharmaceutical Technology and Regulatory Affairs, Faculty of Pharmacy, University of Szeged, Eötvös u. 6, 6720 Szeged, Hungary; falusi.fanni@szte.hu (F.F.); berko.szilvia@szte.hu (S.B.); gasparne.kovacs.anita@szte.hu (A.K.)

**Keywords:** foam stability, hyaluronic acid, xanthan gum, spreadability, rheology

## Abstract

Foams are increasingly popular in the field of dermatology due to their many advantages such as easy spreading, good skin sensation, and applicability in special skin conditions. One of the critical points of foam formulation is the choice of the appropriate stabilizing ingredients. One of the stability-increasing strategies is retarding the liquid drainage of liquid films from the foam structure. Therefore, our aim was the application of different hydrogel-forming polymers in order to retain the stabilizing liquid film. Dexpanthenol and niacinamide-containing foams were formulated, where xanthan gum and hyaluronic acid were used as foam-stabilizing polymers. Amplitude (LVE range) and frequency sweep (G’, G”, tanδ, and frequency dependency) were applied as structure- and stability-indicating rheological parameters. The rheological data were compared with the results of the cylinder method, microscopical images, and the spreadability measurements. The application of the gel-forming polymers increased the stability of the dermal foams (increased LVE range, G’ values, and decreased frequency dependency). These results were in correlation with the results of the cylinder and spreadability tests. It was concluded that in terms of both foam formation and stability, the combination of xanthan gum and dexpanthenol can be ideal.

## 1. Introduction

The use of dermal foams has become widespread in both the pharmaceutical and cosmetic fields. Foams are colloidal systems in which gas bubbles are dispersed in a solid or liquid dispersion medium and the two phases are separated by a solid wall (solid foams) or a liquid lamella (liquid foams). The lamellae can adhere to each other to form a foam. Dermal foams are liquid foams and preferred pharmaceutical products which are used to improve wound healing and treat chronic dermatological diseases and are also used in the pediatric field. They can be considered as special delivery systems due to their many advantages, such as convenient application on extensive or hairy skin surfaces [1]. In addition, their high rate of expansion allows large skin surfaces to be covered rapidly. The bulk liquid, which is stored in a container, is unsaturated or saturated for the active substance used. With the use of an appropriate foaming pump head, foams are produced by adding air to the bulk liquid. The volatile components are rapidly eliminated from the applied foam, resulting in supersaturation. For the active substance, after the foam starts to decay, a supersaturated thin liquid film (lamella) is formed, from which penetration starts at high speed due to the propulsive forces in the system. The fields of application require that the foam remains stable for a sufficient period of time to achieve a good spreading and the desired therapeutic effect, which is also beneficial for improving patient compliance.

In order to find the most suitable components and formulation strategies to ensure the foam stability, the main mechanisms that cause the foam decay must be clarified. The four main mechanisms are drainage, coalescence, Ostwald ripening, and bursting of bubbles [2]. Briefly, drainage is when the liquid drains off owing to gravity. The Plateau borders between the bubbles allow the liquid to flow down through. Foams with a high liquid content can show very round bubbles. As the liquid starts to go through the foam, the bubbles take a polyhedral shape. As a result, the foam becomes unstable because the liquid is crucial to stabilize the lamellas of each bubble. The occurrence of coalescence is the result of surface tension (SFT). The bubbles get close to each other; afterwards, they conjoin and form a single bubble. Ostwald ripening is a consequence of thermodynamical instability between bubbles. In bigger bubbles, the pressure is lower and the small bubbles can dissolve in the solution and re-deposit to form large bubbles. Finally, bursting causes the air to eliminate from the foam bubbles as the liquid film is ruptured.

**Figure 1:**
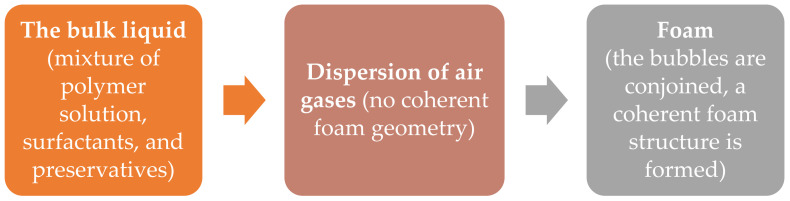
Stages of the foam formation.

Foam formation involves three main stages [3], which are represented in Figure 1.

Foaming techniques can be grouped into physical, chemical, and biological foaming [4]. The physical foaming techniques of mixing and foam formation by foam pumps are discussed in our article. These two techniques are the easiest and the most environmentally friendly methods. During the use of the mechanical stirrer, the turbulence causes air to diffuse into the liquid medium, thus allowing the foam to form. Among dermal foams, the propellant-free pump formulations are more favored. The propellant-free foam pump is easy for patients to use, and it is an environmentally friendly method as the liquid is mixed with the air, resulting in foam generation. The bulk liquid is contained in a liquid dosing chamber. The foam pump device also includes an air dosing chamber. The air from the air dosing chamber and the bulk liquid from the dosing chamber are moved through an uptake tube.

Foam stability is determined by a combination of dynamic and static factors. Forces between the interfaces of two bubbles determine the stability of the liquid film that separates them [4]. The key objective when stabilizing foams is to stop destabilizing mechanisms. Studies over the past decade indicate that many strategies can be used to stabilize foams. These methods may include the use of surfactants, photosurfactants [5], proteins [6], polyelectrolytes [7], and other gel-forming polymers. They can be usually used in combination to achieve the desired stability. The most common combination is the simultaneous use of polymer and surfactant, which are commonly used as foaming agents [8,9]. On the one hand, if higher elasticity can be reached by adding a high concentration of the foaming agent, then the bubbles have to work against the increased interfacial viscoelasticity. This mechanism can provide an energy barrier that could prevent the early shrinkage of the bubbles [10]. Moreover, polymers can increase the viscosity and elasticity of the liquid films, which can greatly contribute to delaying the break-up of lamellae. On the other hand, surfactants can reduce the mobility of the bubbles when adhering to the liquid–gas interface, which delays bubble formation fluctuations. In our research, we used surfactants and gel-forming polymers whose effects on the skin are already recognized, but their effects on foam stability are not known. Regarding surfactants, although ionic surfactants are effective foam stabilizers, they are known to be skin irritants [11] and the use of non-ionic surfactants is therefore recommended, especially when inflamed areas need to be treated. Our research has been conducted to investigate the effect of two popular polymers on foam stability: xanthan gum (XANT) and hyaluronic acid (HA). Xanthan gum is a widely used excipient in pharmaceutics [12,13]. It is one of the only naturally derived thickeners commonly used in dermal preparations. Accordingly, it has the ability to lock in water to help maintain skin hydration. The hyaluronic acid used in the formulae is derived from natural sources and has moisturizing and water-retaining properties. Hyaluronic acid not only promotes skin hydration but also plays a crucial role in wound healing because it has an anti-inflammatory effect and aids tissue regeneration [14,15].

Our work aimed to formulate stable foam formulations containing two potential dermatological active ingredients (API) (dexpanthenol, DEX and niacinamide, NIA), for which two different liquid film stabilizing polymers (namely xanthan gum and hyaluronic acid) were chosen. The formation of foam was analyzed by rheological amplitude sweep test (linear viscoelastic (LVE) range), surface tension, microscopical investigation, and cylinder test. The stability of the foams was investigated through the rheological frequency sweep test and the spreadability test.

The application of the gel-forming polymers improved the stability of dermal foams (wider LVE range, higher G’ values, and decreased frequency dependency). The rheological results were in correlation with the results of the cylinder and spreadability tests. With the applied methods, the ideal combination and composition can be selected.

## 2. Results and Discussion

In the first part of the work, the foam formation ability of the composition was analyzed using surface tension measurement, macroscopic foam stability (foam expansion, FE; foam volume stability FVS), microscopical, and rheological investigations. In this section, two physical foam-forming techniques were compared. Mechanical stirring represents the foam formation more realistically, the duration of the foam formation is more traceable, while the propellant-free pump devices imitate the real conditions of the application.

In the second part of the work, the structure and the applicability of the foams formed by the pump (Figure S1) were evaluated using rheological and texture analyzer technics.

### 2.1. Foam Formation Ability

The surface tension of liquids significantly affects the dispersibility of air, the size [16,17], and the stability of the bubbles formed [18]. The surface tension of the bulk liquids was determined using the pendant drop method. The surface tension of the API-free (AF) and the polymer-free (PF) solution was 27.54 mN/m; compared to this, the addition of polymers caused an increase, i.e., their surfactant inactivity prevailed (Table 1). When dexpanthenol was added to the formulation, a decrease in surface tension was observed in the polymer-free and xanthan gum-containing formulations, suggesting that dexpanthenol may be contributing to the interfacial formation, whereas niacinamide only altered the polymer-free formulations and increased the surface tension in a similar way to polymer-containing formulations. The results suggest (Table 1) that dexpanthenol-containing formulations have the most favorable surface tension for foam formation, even with xanthan gum which increases the surface tension, lower surface tension can be measured to compensate for its surface inactivity.

The lower surface tension may lead to a significantly higher foam expansion (FE). However, the FE-values are more significant in polymer-free compositions, with values above 170%, while in polymer-containing compositions, it is usually below 150%. This is due to the fact that the amount of air emulsified in the foam with mechanical stirring depends significantly on the viscosity of the mixed bulk liquid, and in our case, polymer-containing bulk liquids are considered to be more viscous. Comparing the effect of the two polymer-containing foams on expansion, no distinct correlation can be found.

Regarding foam volume stability, the macroscopic tests confirmed that polymer-free systems collapsed rapidly (FVS values ranging from 11 to 15%), while for polymer-containing systems, xanthan-containing foams maintained their foam volume after 30 min, although a 5–10% volume decrease was observed for HA-containing compositions. Although the addition of the polymer to the bulk liquid increases the viscosity and elasticity, which leads to a more difficult foam formation, the thickness and stability of the lamellae surrounding the bubbles after foam formation are increased [19]. A common example is the polymer-free and xanthan gum samples containing dexpanthenol, where due to the favorable surface tension, the foam formation is the greatest in the case of the polymer-free sample (FE = 180%), while the polymer-containing foam has a much lower value (FE = 114%). By contrast, the parameter indicating their stability (FVS) is much more favorable in the sample containing xanthan gum (100% vs. 15%).

**Figure 2:**
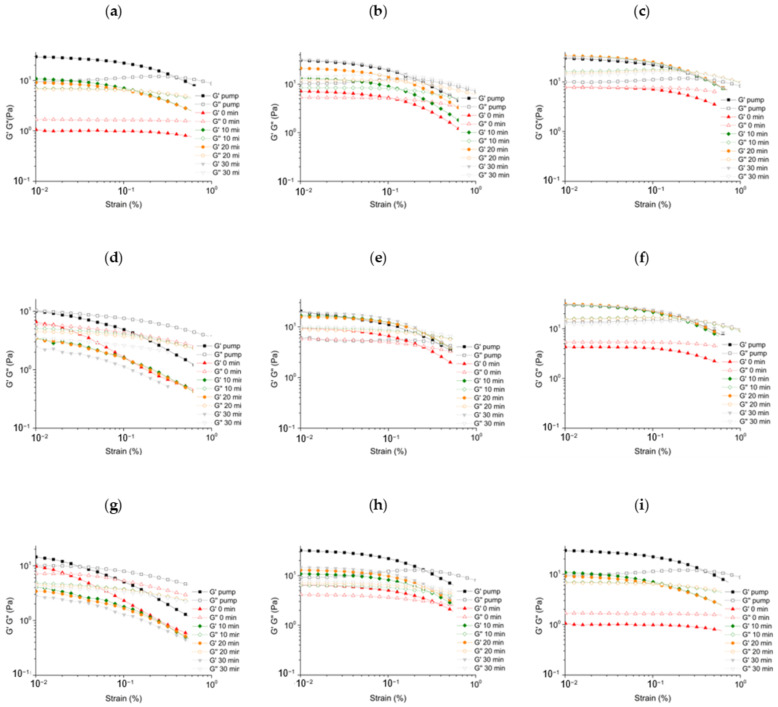
The amplitude sweep of the formed foams (**a**) without polymer; (**b**) with XANT; (**c**) with HA.; (**d**) DEX: without polymer; (**e**) DEX with XANT; (**f**) DEX with HA; (**g**) NIA without polymer; (**h**) NIA with XANT; (**i**) NIA with HA.

During rheological investigations, the linear viscoelastic range of the formed foams (Figure 2) was determined, and measurements were performed with pump-formed foams, and also foams prepared with mechanical stirrer after preparation (0 min) and 10, 20, and 30 min. With amplitude sweep, the mechanical resistance of the foams can be determined and the formation of the foam, namely the formation of the foam structure can be identified. The interfacial interactions between bubbles and lamellae induced a coherent structure, which can be characterized by the storage (G’) and loss (G”) moduli on the rheological curves for the elastic and viscous properties.

In our case, for compositions defined as real foam, when the coherent structure is formed, the G’ modulus was higher than the G” modulus, and it was constant up to a given strain value, depending on the strength of the foam structure. The end of linearity, and the associated strain value, represent the resistance of the structure to deformation; the higher the strain value, the more resistant the structure is. The coherent foam structure is weak, and the viscoelastic range of our formulated foams ends under 1% strain (Figure 2). These results are similar to values indicated in previous literature on topically applied foams [20]. We could also observe that samples from the aged foams were rheologically divergent (Table S1). In some cases, water leaking out of the lamellae means a decrease in the fluidity of the lamellae, so the viscoelastic range is extended. This behavior could be observed in most foams; however, it should be added that in polymer-free formulations, the measurements were performed on a foam that had already shrunk significantly (below 15% FVS-value). In several cases, the coherent structure was no longer measurable, and the classical foam structure disappeared (PF-AF at 30 min; DEX-PF at 30 min). In these cases, the G” modulus was higher than the G’ modulus, and no linearity was observed, so the end of the viscoelastic range in the investigated range was not detectable.

The foams formed with mechanical stirrer and the foams formed with pumps were also compared throughout the rheological studies. The linear viscoelastic range of the foams generated by the mechanical stirrer is most similar to the initial rheological values of the non-aging foam, showing a highly rapid structural breakdown (γ_LVER_ < 0.3%). In general, the viscoelastic range of polymer-free systems breaks down earlier, resulting in a more fragile structure when produced by both mechanical stirrer and pump.

**Figure 3:**
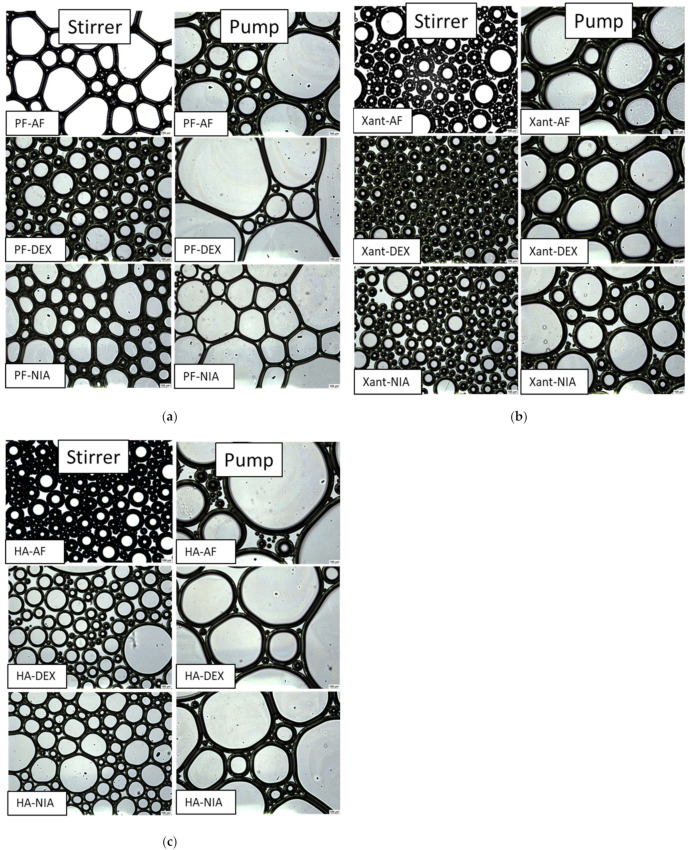
The comparison of the structure of foams produced by the pump and mechanical stirring: (**a**) polymer-free foams; (**b**) xanthan gum-containing foams; (**c**) hyaluronic acid.

The structure of foams produced by mechanical stirrer and pump is illustrated by microscopic images (Figure 3). Foams produced with a pump exhibit a more heterogeneous structure, meaning that there is a variety of smaller and larger bubbles, whereas the size of the bubbles was smaller and more uniform in systems produced with a mechanical stirrer.

Microscopic images provide the opportunity to study the film or lamellae that bound the bubbles. In general, a thicker wall structure was observed in polymer-containing foam systems, which was more noticeable in pump-formed foams. When comparing the two polymers, the film thickness may have been greater for xanthan gum-containing compositions, and the amount of liquid bound by the polymer in the interfacial layer may have been more significant.

Microscopic images enable the determination of the bubble sizes as well as the characterization of the wall thickness of the lamellae around the bubbles. However, several authors [21,22] and our own experience point out that conventional microscopic sectioning is difficult due to the dynamic behavior of foams and their weak structure; therefore, occasionally no conclusions can be drawn. In order to avoid this, our work compared only the differences in bubble size (monodisperse or heterodisperse) and wall thicknesses for microscopic evaluations, without detailed statistical measurements. For a more accurate characterization of the structure, we therefore subsequently used methods that provide well-reproduced information on a large sample volume (rheology and texture analyzer)

### 2.2. Characterization of the Structure of Foams Generated by Pumps

Dermal foams are usually formed using propellent-free pump devices, and therefore it was considered necessary to investigate the structure of these foam systems in more detail.

**Figure 4:**
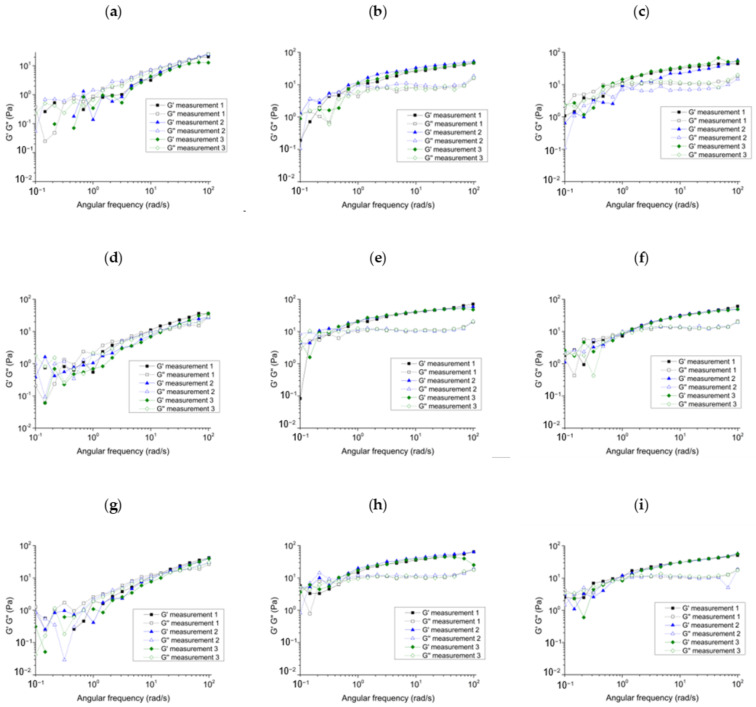
The frequency sweep of the formed foams (**a**) without polymer; (**b**) with XANT; (**c**) with HA.; (**d**) DEX: without polymer; (**e**) DEX with XANT; (**f**) DEX with HA; (**g**) NIA without polymer; (**h**) NIA with XANT; (**i**) NIA with HA. Three parallel measurements are presented.

The formed coherent gel structure was investigated with frequency sweep (Figure 4). The value of the G’ and G” moduli, their relationship to each other (tanδ), and their frequency dependence provided information about the strength and stability of the formed foam structure (Table 2). For polymer-free systems, the moduli showed much lower values, the storage modulus was lower than the loss modulus, and the tanδ values were below 1, which means that the coherent structure did not characterize the formed foam. The significant frequency dependence (high slope value) of the polymer-free samples also indicated instability. The addition of polymer in the system showed an increase in elasticity, an increase in G’ modulus values, and an increase in tanδ and frequency dependence. The addition of the polymer referred to the strengthening of the coherent structure. In the API-free systems, the two polymers resulted in similar changes in rheological parameters, with no significant difference. However, it is remarkable that dexpanthenol together with xanthan gum significantly increased the elasticity and stability of the foam. The G’ modulus increased from 11.2 Pa to 20.3 Pa after the addition of dexpanthenol, while tanδ decreased from 0.30 to 0.26, similarly, the frequency-dependent slope decreased from 0.50 to 0.33. This formulation had a very low surface tension value which indicates the xanthan gum and the dexpanthenol together can modify the bubble-liquid film interface. This modification may change the mechanical behavior of the foam based on the results presented earlier, which also suggests that the combination of polymer and dexpanthenol strengthens the effect of each other.

Commercially available dermally applied foams can have very different rheological properties [20]. In our research, the rheological parameters were used to optimize the composition in order to select the appropriate formulation with the most favorable stability and structure. The combination of dexpanthenol and xanthan gum is also indicated as the optimal formulation in this respect.

The spreadability of the foams can appear to be critical for application and for this purpose, a texture analyzer was used to determine the firmness values, which are characterized by the maximum strength of the force-distance curve required to spread the foam to a thickness of 1 mm (firmness). The results suggest that polymer-free foams with no elastic properties could be spread with much lower forces, as predicted from the rheological properties. No clear correlation was observed between the two foam systems containing different polymers in terms of spreading. In the absence of a similar study of foams in previous literature, we compared the values obtained with the rheological parameters in our research. With the texture analyzer, the foams did not show as much difference as with the rheological parameters, which means that the rheological parameters can provide a more accurate indication of the effect of both polymers and active ingredients on the structure.

## 3. Conclusions

The purpose of our work was to formulate a dermal foam in which the ingredients are present in quality and quantity that results in a foam structure with adequate mechanical strength and stability. Two potential dermal agents (dexpanthenol, niacinamide) were used with the aim of selecting an ideal polymer.

The results proved that the addition of the polymer resulted in a much more stable and better structured foam. The xanthan gum provided more favorable rheological properties and more stable foam systems. In terms of both foam formation and stability, the combination of xanthan gum and dexpanthenol proved to have the most beneficial properties, making it an ideal combination.

## 4. Materials and Methods

### 4.1. Materials

Kolliphor RH 40 was provided by BTC Europe GmbH-A BASF Group Company (Burgbemheim, Germany). Labrasol ALF was supplied by Azelis Hungary Ltd. (Budapest, Hungary), Xantural^®^ 180 was obtained from Biesterfeld GmbH (Budapest, Hungary). Verstatil PC was purchased from Biesterfeld GmbH (Budapest, Hungary). Purified and deionized water was used (Milli-Q system, Millipore, Milford, MA, USA). HyaCare Tremella (1240 kDa) was a product sample from Finecon-Evonik Industries AG (Essen, North Rhine-Westphalia, Germany). Niacinamide was kindly supplied by Biesterfeld GmbH (Budapest, Hungary). D-Panthenol was by DSM Nutritional Products Ltd. (Basel, Switzerland).

### 4.2. Methods

#### 4.2.1. Composition and Preparation of Foam Samples

The exact composition of different foam formulations is shown in Table 3. The type and the concentration of the surfactants and preservative were chosen based on our earlier study [23].

Each formulation contains the same amount of surfactants, preservatives, and solvents. The difference between the formulations is the contained polymer and active ingredient. As the aim is to develop an appropriate foam formula for dermatological use, we were looking for a suitable polymer for transdermal application. Niacinamide and dexpanthenol are the active ingredients used as they contribute to the improvement of the skin’s barrier function.

Phase A contains a mixture of surfactants, which are the main foaming agents of the formulation. Phase B contains the chosen polymers, the solvent, and one of the active ingredients. When preparing Phase B, the polymer was swollen in half the amount of purified water for 1.5 h. In the remaining water, a solution was made with the active ingredient. After the complete dissolution of the active ingredient and swelling of the polymer, the two solutions were then mixed. The first step was the addition of Phase C to Phase A. Lastly, Phase B was mixed with the mixture of Phases A and C. After the preparation of bulk liquids, the samples were kept in well-sealed jars and propellant-free foam pumps until the start of the investigations. To compare the effect of the two production methods, the tested foams were generated with a propellant-free foam pump. In the other method of preparation, 30 g of bulk liquid was stirred with the IKA stirrer for 5 min at 2000 rpm, before each investigation.

#### 4.2.2. Macroscopic Characterization of Foams

In order to develop appropriate compositions, it is important to identify test methods for investigating the stability of foams. Foam stability is the parameter that indicates the period of time until which the foam can maintain its initial properties. These can be tested to provide data on foamability and foam stability. This method has only been performed with foams produced with mechanical stirring. First, 30 g bulk liquid was stirred for 5 min, then a glass graduated cylinder was filled up with the foam. The initial foam volumes were recorded, and after 30 min the aged foam volumes.

The parameters can be calculated using the following formulae [24]:

(1) $$\mathrm{FVS}\left(\%\right)=\frac{V\left(\mathrm{foam},\;30\;\min\right)}{V\left(\mathrm{foam}\right)}\cdot 100\%$$

where V(foam,30 min) is the foam volume after 30 min (mL).

(2) $$\mathrm{FE}\left(\%\right)=\frac{V\left(\mathrm{foam}\right)-V\left(\mathrm{formulation}\right)}{V\left(\mathrm{formulation}\right)}\cdot 100\%$$

where V(formulation) is the volume of the formulation (mL) required to produce V(foam) (mL) [24].

The cylinder method can be used to determine the foam volume stability (FVS, %) and foam expansion (FE, %), as it is an easy and convenient method [24].

#### 4.2.3. Spreadability (Texture Analyzer)

With texture analyzer, dermal application of any semi-solid dosage forms can be modeled. The instrument measures the forces required to spread the product on the skin.

The spreadability of the foams was examined with a TA.XT plus Texture Analyzer (Stable Micro Systems Ltd., Vienna Court, Lammas Road, Godalming, Surrey, UK. GU7 1YL) using a TTC Spreadability Rig, which includes a 90° cone-shaped male probe and a precisely fitting female cone-shaped, Perspex product holder. When a trigger force of 1 g was achieved, the male probe proceeded to penetrate the sample at a test speed of 10 mm/s until a gap of 1 mm. The force to penetrate the sample increased during this time. The tested foam flowed outward at 45° between the surfaces of male and female cones and the ease of spreading indicated the degree of spreadability. The maximum force value in the force-distance graph is a measure of firmness that characterizes the spreadability of the sample.

#### 4.2.4. Microscopic Characterization of Foams and Image Analysis

The investigation of the microscopically observable properties of foams was carried out with Leica DM6 B Fully Automated Upright Microscope System (Leica Biosystems GmbH, Wetzlar, Germany). The structure of the foams can be observed with microscopic images. The roundness, size, size distribution of bubbles, and the thickness of lamellae between two bubbles can provide paramount information on foam stability. The structure of the foam was analyzed immediately after the generation of foams through the light microscope.

#### 4.2.5. Determination of the Surface Tension

The surface tension affects the foam stability. Measuring the surface tension is of major importance because the lower surface tension assists the bubble formation and the higher surface elasticity helps to maintain the stability of the foam lamellae [25]. The surface tension of bulk liquids was measured with OCA 20 Contact Angle System (Dataphysics Instruments GmbH, Filderstadt, Germany) by analyzing the shape of the pendant drop. The pendant drop was created at the end of a needle with a predefined inner and outer diameter in a vertical position. The contour of the droplet was detected by the SCA20/22 software V.5.0.41 module using the Young–Laplace equation. The camera was rotated by 90° for a better resolution, which allowed the software to get a larger screenshot and a more accurate contour analysis, in which case the camera image detected the hanging droplet from left to right. As the instrument detected the contour of the pendant drop, it calculated the surface tension from it. The average of 5 parallel measurements was used for the evaluation.

#### 4.2.6. Oscillometric Measurements

Studying the structure of foams has always been problematic, as the structure of foams is easily friable and often contains large bubbles. Oscillometric methods (amplitude sweep, frequency sweep) can be used to gain more insight into the mechanical properties of foams such as the elasticity of the film, and thus the stability of the foam. The rheological properties of foams were studied at 25 °C with an Anton Paar Physica MCR302 Rheometer (Anton Paar, Graz, Austria). The measuring device was of the parallel plate type (diameter 50 mm, gap height 2 mm). Each measurement was performed immediately after the generation of foam samples or after a given storage time (10, 20, and 30 min).

#### 4.2.7. Amplitude Sweep

The foams were analyzed through amplitude sweeps [20], at an increase of the strain value from 0.1% to 100%, and at a constant angular frequency of 10 rad/s. The linear viscoelastic (LVE) range was determined by the RheoCompass™ software v.1.25 (Anton Paar, Graz, Austria). We selected a 3% tolerance range of deviation for G’ around the plateau value for the determination of the LVE range. The results of the measurement of the LVE range were used for the setting of the frequency sweep tests, and the LVE range can give information on the formation of foam structure.

#### 4.2.8. Frequency Sweep

Storage modulus (G’), loss modulus (G”), loss factor (tanδ), and the slope of the curve (frequency-dependency) were determined [20] over the angular frequency range from 0.1 to 100 rad/s. The applied strain value (0.1%) was in the range of the linear viscoelasticity of the foams. Three parallel measurements were carried out.

## Figures and Tables

**Table 1:** The results of the surface tension, foam expansion, and foam volume stability measurements.

Sample	SFT (mN/m)	FE (%)	FVS (%)
PF-AF	27.54 ± 0.11	172.2 ± 15.8	14.3 ± 1.8
XANT-AF	29.20 ± 0.06	134.4 ± 1.9	100.0 ± 0.0
HA-AF	28.78 ± 0.33	125.6 ± 3.8	94.6 ± 0.1
PF-DEX	24.32 ± 1.18	180.0 ± 0.0	15.1 ± 0.7
XANT-DEX	24.00 ± 3.85	114.4 ± 1.9	100.0 ± 0.0
HA-DEX	28.40 ±0.30	144.4 ± 1.9	88.2 ± 0.7
PF-NIA	29.20 ± 0.22	183.3 ± 0.0	11.8 ± 0.0
XANT-NIA	29.49 ± 0.08	126.7 ± 3.3	100.0 ± 0.0
HA-NIA	29.31 ± 0.01	113.3 ± 3.3	91.9 ± 1.3

**Table 2:** Results of the frequency sweep measurement and the spreadability of the foam formed by the pump.

Sample	Rheology				Spreadability
	G’ at 10 rad/s (Pa)	G” at 10 rad/s (Pa)	tanδ at 10 rad/s (-)	Slope	Firmness (mN)
PF-AF	3.86 ± 0.61	6.60 ± 0.87	1.76 ± 0.46	1.89 ± 0.33	120.9 ± 5.0
XANT-AF	29.09 ± 3.81	8.71 ± 1.24	0.30 ± 0.03	0.51 ± 0.09	229 ± 6.7
HA-AF	29.73 ± 6.29	9.83 ± 2.38	0.33 ± 0.01	0.53 ± 0.03	291.9 ± 5.8
PF-DEX	8.51 ± 2.32	9.38 ± 0.69	1.14 ± 0.23	0.85 ± 0.08	127.9 ± 11.3
XANT-DEX	40.35 ± 0.74	10.63 ± 0.38	0.26 ± 0.01	0.34 ± 0.03	275.8 ± 4.9
HA-DEX	30.60 ± 1.27	15.62 ± 3.76	0.45 ± 0.02	0.56 ± 0.04	271.0 ± 8.9
PF-NIA	9.32 ± 1.65	11.04 ± 1.35	1.19 ± 0.06	1.30 ± 0.46	145.9 ± 8.5
XANT-NIA	38.02 ± 2.58	11.13 ± 0.49	0.29 ± 0.01	0.42 ± 0.03	254.2 ± 6.3
HA-NIA	30.96 ± 0.26	11.29 ± 0.62	0.36 ± 0.02	0.47 ± 0.07	258.6 ± 14.4

**Table 3:** Composition of the investigated formulations.

	Polymer Free Compositions			Xanthan Gum-Containing Compositions			Hyaluronic Acid-Containing Compositions		
Components	PF-AF	PF-DEX	PF-NIA	XANT-AF	XANT-DEX	XANT-NIA	HA-AF	HA-DEX	HA-NIA
Phase A									
Labrasol ALF /surfactant/	+	+	+	+	+	+	+	+	+
Kolliphor RH40 /surfactant/	+	+	+	+	+	+	+	+	+
Phase B									
Xanthan gum (Xant.) /polymer/	-	-	-	0.2%	0.2%	0.2%	-	-	-
Hyaluronic acid (HA) /polymer/	-	-	-	-	-	-	0.2%	0.2%	0.2%
Purified water /solvent/	+	+	+	+	+	+	+	+	+
Dexpanthenol /active ingredient/	-	5%	-	-	5%	-	-	5%	-
Niacinamide /active ingredient/	-	-	5%	-	-	5%	-	-	5%
Phase C									
Phenoxyethanol /preservative/	+	+	+	+	+	+	+	+	+

+ the component is in the formulation, - the component is not in the formulation.

## Data Availability

Data are contained within the article or Supplementary Materials.

## Supplementary Materials

The following supporting information can be downloaded at: https://www.mdpi.com/article/10.3390/gels8070413/s1, Table S1: Calculated LVE range of the foams; Figure S1: Photos of the different foams. (a) PF-AF, XANT-AF, HA-AF; (b) PF-DEX, XANT-DEX, HA-DEX, and (c) PF-NIA, XANT-NIA, HA-NIA.

## Abbreviations

AF	API-free
API	active ingredient
DEX	dexpanthenol
FE	foam expansion
FVS	foam volume stability
HA	hyaluronic acid
NIA	niacinamide
PF	polymer-free
STF	surface tension
XANT	xanthan gum
