# Supplementary material for: Application of Xanthan Gum and Hyaluronic Acid as Dermal Foam Stabilizers
Source: Gels. 2022 Jun 30;8(7):413. doi: 10.3390/gels8070413 (PMC9321585; doi:10.3390/gels8070413)
Supplement: Supplementary file 1 [file gels-08-00413-s001.zip › gels-1771812-supplementary.pdf]

gels

MDPI

Article

Application of Xanthan Gum and Hyaluronic Acid as Dermal Foam Stabilizers

Fanni Falusi, Szilvia Berkó, Anita Kovács and Mária Budai-Szűcs *

Institute of Pharmaceutical Technology and Regulatory Affairs, Faculty of Pharmacy, University of Szeged, Eötvös u. 6, 6720 Szeged, Hungary; falusi.fanni@szte.hu (F.F.); berko.szilvia@szte.hu (S.B); gasparne.kovacs.anita@szte.hu (A.K.)
* Correspondence: budai-szucs.maria@szte.hu; Tel.: +3662545573

Supplementary material

Table S1. Calculated LVE range of the foams.

Sample		γ_{LVER} (%)				
		mechanical stirrer				pump
API	Polymer	0 min	10 min	20 min	30 min	
AF	PF	0.20	0.49	0.21	ND	ND
	Xant	0.23	0.24	0.26	0.69	0.21
	HA	0.16	0.79	0.69	0.66	0.23
DEXP	PF	0.28	0.18	0.45	ND	0.15
	Xant	0.32	0.49	0.46	0.36	0.18
	HA	0.16	0.82	0.58	0.70	0.23
NIA	PF	0.19	0.18	0.16	0.45	0.21
	Xant	1.21	0.69	0.80	0.42	0.32
	HA	0.11	0.21	0.27	ND	0.32

ND: not detectable.

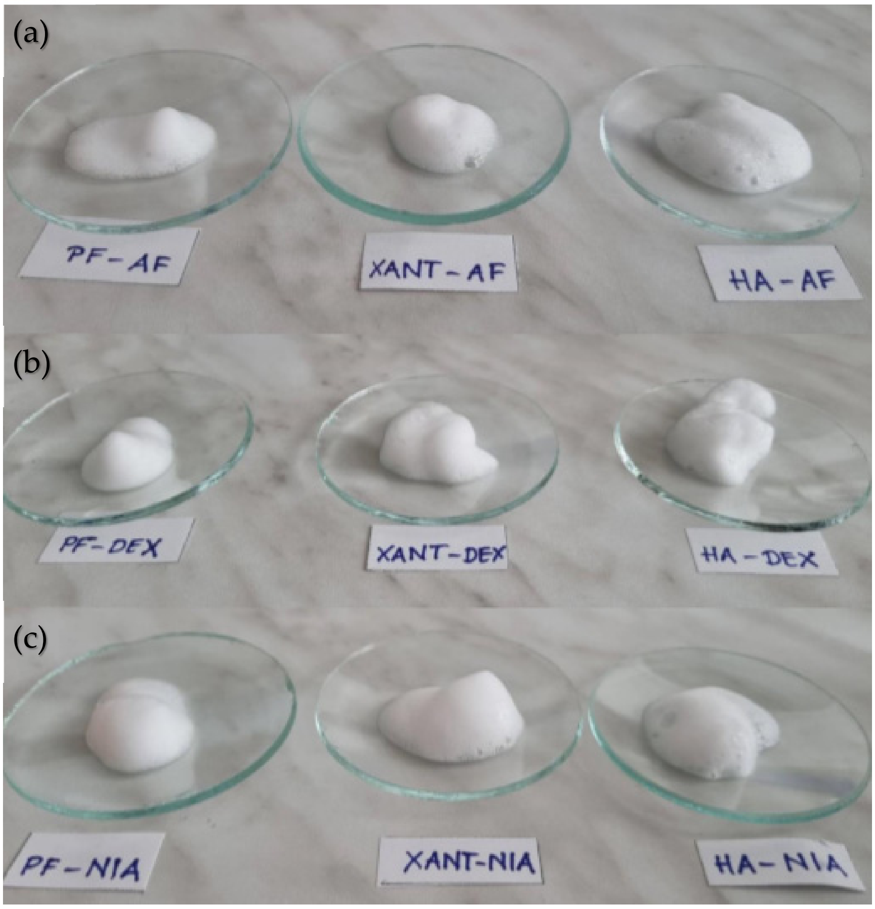

Figure S1. Photos of the different foams (**a**) PF-AF, XANT-AF, HA-AF; (**b**) PF-DEX, XANT-DEX, HA-DEX, and (**c**) PF-NIA, XANT-NIA, HA-NIA.
